# Spiders in rice-paddy ecosystems shift from aquatic to terrestrial prey and use carbon pools of different origin

**DOI:** 10.1007/s00442-020-04601-3

**Published:** 2020-01-30

**Authors:** Nico Radermacher, Tamara R. Hartke, Sylvia Villareal, Stefan Scheu

**Affiliations:** 1 J. F. Blumenbach Institute of Zoology and Anthropology, University of Göttingen, Göttingen, Germany; 2 Crop and Environmental Sciences Division, International Rice Research Institute (IRRI), Los Baños, Philippines; 3 Centre of Biodiversity and Sustainable Land Use, University of Göttingen, Göttingen, Germany

**Keywords:** Rice field, Stable isotopes, Generalist predators, Biological control, Rice insect pest

## Abstract

Spiders are important bio-control agents of rice insect pests such as plant- and leafhoppers. To investigate temporal changes in spider prey and variations in prey due to landscape structure around rice fields, carbon and nitrogen stable isotopes of rice field arthropods were analysed over three consecutive sampling dates during the rice cropping season. Initial isotope composition of gnats and midges emerging from submersed rice fields indicates a larval algae diet, while later values suggest a switch to rice-derived carbon. Initial δ^13^C values of plant- and leafhoppers were higher in fields of rice-heterogeneous landscapes, indicating migration from source populations feeding on C4 grasses into rice fields; later, their δ^13^C values approached those of rice. Isotope values of web-building and cursorial spiders in the earliest samples indicate aquatic gnat and midge prey. The later shift toward terrestrial herbivore prey was more pronounced for small than for larger species and in rice paddies near permanent vegetation, indicating use of prey from the surrounding landscape. The results suggest that rice field spiders are supported by three different carbon pools: (1) aquatic carbon originating from algae and (2) legacy carbon from previous growing cycles, both incorporated via between-season predation on gnats and midges, and (3) carbon from the current rice season incorporated via herbivore prey. In conclusion, fostering aquatic midge and gnat larvae, e.g. via mulching, and integrating rice fields into rice-heterogeneous landscapes likely strengthens biological control of pest species in rice paddies by supporting high populations of spiders between cropping seasons.

**Electronic supplementary material:**

The online version of this article (10.1007/s00442-020-04601-3) contains supplementary material, which is available to authorized users.

## Introduction

Rice (*Oryza sativa*) is one of the most important staple crops in the world, providing food to almost half of the world population and making up 27% of the world populations’ caloric uptake (FAO/UN 2004). With an ever-growing human world population, food security, therefore, is of outmost importance for rice-growing countries of temperate and tropical regions, including the Philippines. The green revolution led to agricultural intensification promoting high-input cultivation practices, such as application of mineral fertilisers and pesticides (Savary et al. 2012). High nitrogen input promotes sap-sucking insect herbivores; while insecticides may reduce not only pest species but also their natural enemies, such as spiders, thereby reducing biological control of planthoppers (Delphacidae) and leafhoppers (Cicadellidae) resulting in yield losses (Kiritani 1979; Settle et al. 1996; Rashid et al. 2017).

With more than 40,000 species, spiders (Araneae) are diverse and ubiquitous predators, with the majority of species following a generalist foraging mode and thereby having a broad spectrum of prey organisms (Foelix, 2011; Riechert and Lockley 1984). Their ability to hunt in a variety of habitats in combination with high abundance positions spiders as potentially effective biocontrol agents (Riechert 1999; Symondson et al. 2002; Wise 1993). Dispersal by running and ballooning allows spiders to colonise agricultural fields soon after disturbance due to agricultural practices such as ploughing and seed sowing. This applies in particular to tropical arable systems with multiple cropping cycles per year and asynchronous planting practice (Marc et al. 1999; Sunderland and Samu 2000). In fact, spiders are among the most abundant arthropod predators in tropical rice ecosystems and assumed to contribute to the control of pest species such as plant- and leafhoppers (Heong et al. 1991, 1992; Sigsgaard 2007).

With the ability to capture prey of different feeding guilds, including herbivores and detritivores, spiders may play an important role soon after planting rice fields when herbivore populations still are low. Generalist predators in agricultural systems such as spiders may link above-ground herbivore and below-ground detrital systems using prey of both of these systems (Scheu 2001; Snyder and Wise 2001; Wise et al. 2006). Von Berg et al. (2010) showed increased predation rates on aphids in wheat fields by carabid and staphylinid beetles due to mulching (applying dead organic matter like crop residue to the field), and Miyashita et al. (2003) found reduced spider abundance when the emergence of detritivores from the soil was inhibited in forests. Settle et al. (1996) demonstrated that, besides avoidance of pesticides, availability of alternative prey from the detrital system is critical for enhancing and maintaining high abundances of generalist predators in rice fields.

The analysis of natural variations in stable isotope ratios allows insight into the trophic position and basal food resources of animals in aquatic as well as terrestrial systems (Minagawa and Wada 1984; Post 2002; Scheu 2002; Potapov et al. 2018). For example, stable isotope analysis (SIA) detected prey shifts of spiders following increased detritivore abundances after adding detritus to vegetable gardens (Wise et al. 2006). SIA of rice field arthropods suggests that spiders forage on aquatic midges (Chironomidae) early in the rice cropping season before shifting their diet to herbivore plant- and leafhoppers later in the season, but this has only been studied in a single temperate rice field without considering the wider spatial context (Park and Lee 2006). It is known that the structure and composition of surrounding habitats (hereafter, landscape structure) can alter food availability for spiders by providing additional prey from nearby ecosystems (Polis et al. 1997; Hambäck et al. 2016), potentially affecting their function as predators of rice field pest species.

In the present study, we used SIA to investigate the structure of arthropod food webs and the role of spiders as generalist predators in paddy-rice ecosystems. Specifically, we investigated the following hypothesises: (1) Spiders use emerging adult aquatic insects, such as gnats (Ceratopogonidae) and midges early in the rice-growing season, before (2) switching towards herbivore pest species of rice later in the season, and (3) rice-heterogeneous landscapes benefit spiders via increased prey availability from non-rice field habitats, thus raising the contribution of terrestrial prey to spider nutrition and thereby the efficiency of spiders as predators of rice pest species.

## Materials and methods

### Location

The study was set up in Laguna Province, Luzon, Philippines on two pairs of fields investigated in the framework of the interdisciplinary LEGATO project (Settele et al. 2015). The study area in Central Luzon has a dry season from November to April and a wet season with a southwest monsoon from May to October (GRiSP 2013). Monthly mean temperatures in the study area (Los Baños, Laguna Province, Philippines) during the study period are 27.4–28.8 °C (climate-data.org 2019). The area is characterised by intensive irrigated lowland rice cropping alongside other farming systems, including fruit plantations and vegetable gardens. The studied rice fields were located between 121.36° and 121.41° E and 14.11° to 14.18° N at altitudes from 25 to 275 m above sea level. The size of the fields ranged from 820 to 2400 m^2^ (Online Resource 1). Study fields were selected in two different landscape structures. Fields within more heterogeneous landscapes were embedded in a matrix of vegetable gardens, extensively managed agroforests with fruit trees, or small unmanaged forests, shrubs and grassland. The rice field area in this landscape, henceforth referred to as “rice-heterogeneous”, comprised a maximum of 30% rice within 100 m around the focal rice field. Fields within more homogeneous landscape comprised a minimum of 50% rice fields at a distance of 100 m around the focal rice field and were dominated by intensively managed rice monocultures, henceforth referred to as “rice-homogeneous” landscape. Rice-homogeneous and rice-heterogeneous fields were located at least 300 m apart from each other and the distance between the two pairs was > 15 km. The elevation gradient was independent of landscape structure assignments.

### Sampling

Field sampling was conducted during the rainy season from June to August 2012. Samples were taken 13–14, 27–29 and 41–43 days after transplantation of rice seedling into the fields (termed as 2, 4 and 6 weeks after transplantation, respectively). To capture a broad range of rice-field arthropods for SIA, three sampling methods were used at each sampling date: sweep net, dip net and suction sampler. Samples were taken at three locations within each field: margin, halfway between margin and centre, and centre. Sweep netting consisted of 30 beats through the rice canopy per field location with a 30-cm-diameter net. Dip netting employed an 18-cm-diameter net with 800-µm mesh size drawn along a 10-m transect per field location. Suction sampling with a modified leaf blower (Blower-Vac; Arida and Heong 1992) was used to catch arthropods from the lower part of the rice plant and the water surface. Samples were taken from an enclosure of 1-m height covering 0.25 m^2^ surface area placed at the margin, halfway between margin and centre, and centre of the field, continuing as long as arthropods were detected in the enclosure. Captured animals were transferred to plastic bags, killed by freezing at − 20 °C and then stored in 70% ethanol at − 20 °C until sorting and identification of gnats and midges, plant- and leafhoppers and spiders (Stehr 1997; Barrion and Litsinger 1994, 1995). Preservation in ethanol little affects ^15^N/^14^N values in arthropods, but can slightly enrich ^13^C/^12^C values, although this effect is expected to be negligible because the treatment was consistent across all samples (Fabian 1998; Hogsden et al. 2017). Simultaneous to arthropod sampling, three rice plants were collected from the three different locations in the field. Rice plants were oven dried at 60 °C for 48 h, frozen and stored at − 20 °C.

### Stable isotope analysis

Larvae and adults of gnats and midges, plant- and leafhoppers, spiders and rice plants were dried at 60 °C for 48 h. Large spiders such as tetragnathids (Tetragnathidae) were ground entirely and a subsample used for the analysis; while small species were used whole. Samples were transferred into tin capsules which were closed before analysis. SIA was carried out by a combination of an elemental analyser (NA 1110, CA-Instruments, Milano, Italy) coupled with an isotope mass spectrometer (Delta Plus, Finnigan MAT, Bremen, Germany; Reineking et al. 1993). Natural variations in stable isotope ratios were expressed using the δ notation as δ*X* (‰) = (*R*_sample_ − *R*_standard_)/*R*_standard_ × 1000, with *X* representing ^13^C or ^15^N, *R*_sample_ the ^13^C/^12^C or ^15^N/^14^N ratio of the sample and *R*_standard_ the respective ratios of the standard. Vienna Peedee Belemnite limestone and atmospheric nitrogen were used as standards for ^13^C and ^15^N, respectively. Acetanilide (C_8_H_9_NO, Merck, Darmstadt) was used for internal calibration.

### Statistical analysis

Changes in the abundances of individual animal groups (gnats and midges, plant- and leafhoppers, spiders) with sampling date (2, 4, 6 weeks after transplantation) and surrounding landscape structure (non-rice dominated and rice-homogeneous) were analysed for sweep net samples only using generalised linear mixed models. Data for each taxon were examined separately to ensure that they met assumptions. The models were specified with “abundance” as response variable and independent variables “date” and “structure” representing sampling date and surrounding landscape structure, respectively. The response variable was log-transformed and specified with Gaussian distribution for gnats and midges. Negative binomial and Poisson distribution were specified for plant- and leafhoppers. The variables “date” and “structure” were included as fixed factors; whereas, “location” (three sampling locations within the field plot) was included as random factor nested in “field”. Akaike Information Criterion (AIC) was used to compare models; models were simplified by progressively removing non-significant variables to obtain the minimal adequate model. Residual plots of the models were inspected visually for outliers. Differences between means were inspected using Tukey’s HSD test at *p* < 0.05.

Variations in carbon stable isotope ratios of gnats and midges, plant- and leafhoppers and spiders by sampling date were analysed using linear mixed-effects models. The dataset was non-orthogonal because not all species were equally represented at each sampling date. Prior to the analyses, data were inspected for homoscedasticity (Fligner–Killeen test) and normality (Shapiro–Wilk test). The independent variables “date”, “taxon” (taxonomic order), “species” and “structure” were included as fixed factors; whereas, “location” was included as random factor nested in “field”. AIC was used to compare models, which were simplified by progressively removing non-significant variables to obtain the minimal adequate model. Residual plots of the models were inspected visually for outliers. Differences between means were inspected using Tukey’s HSD test at *p* < 0.05.

Relative contributions of gnats and midges, plant- and leafhoppers to the diet of spider species were calculated for each sampling date and field using the Bayesian mixing model FRUITS version 2.1.1 Beta (Fernandes et al. 2014). Fractionation factors including standard deviation per trophic level were set to 0.47 ± 1.23 and 3.41 ± 0.41‰ for carbon and nitrogen, respectively, after Vander Zanden and Rasmussen (2001). Values on the contribution of prey taxa to the diet of spider species were analysed using linear mixed-effects models after arcsine transformation. The dataset was non-orthogonal because not all spider species were equally represented at each sampling date. The independent variables “date”, “spider species” and “structure” were included as fixed factors; whereas, “field” was included as random factor. Data inspection prior to the analysis and model selection was conducted as described above. Statistical analyses were performed in R version 3.3.1 (R Core Team 2016) and the packages nlme (Pinheiro et al. 2019), afex (Singmann et al. 2018) and multcomp (Hothorn et al. 2008). Figures were plotted using the R package ggplot2 (Wickham 2009).

## Results

### Abundance

**Fig. 1:**
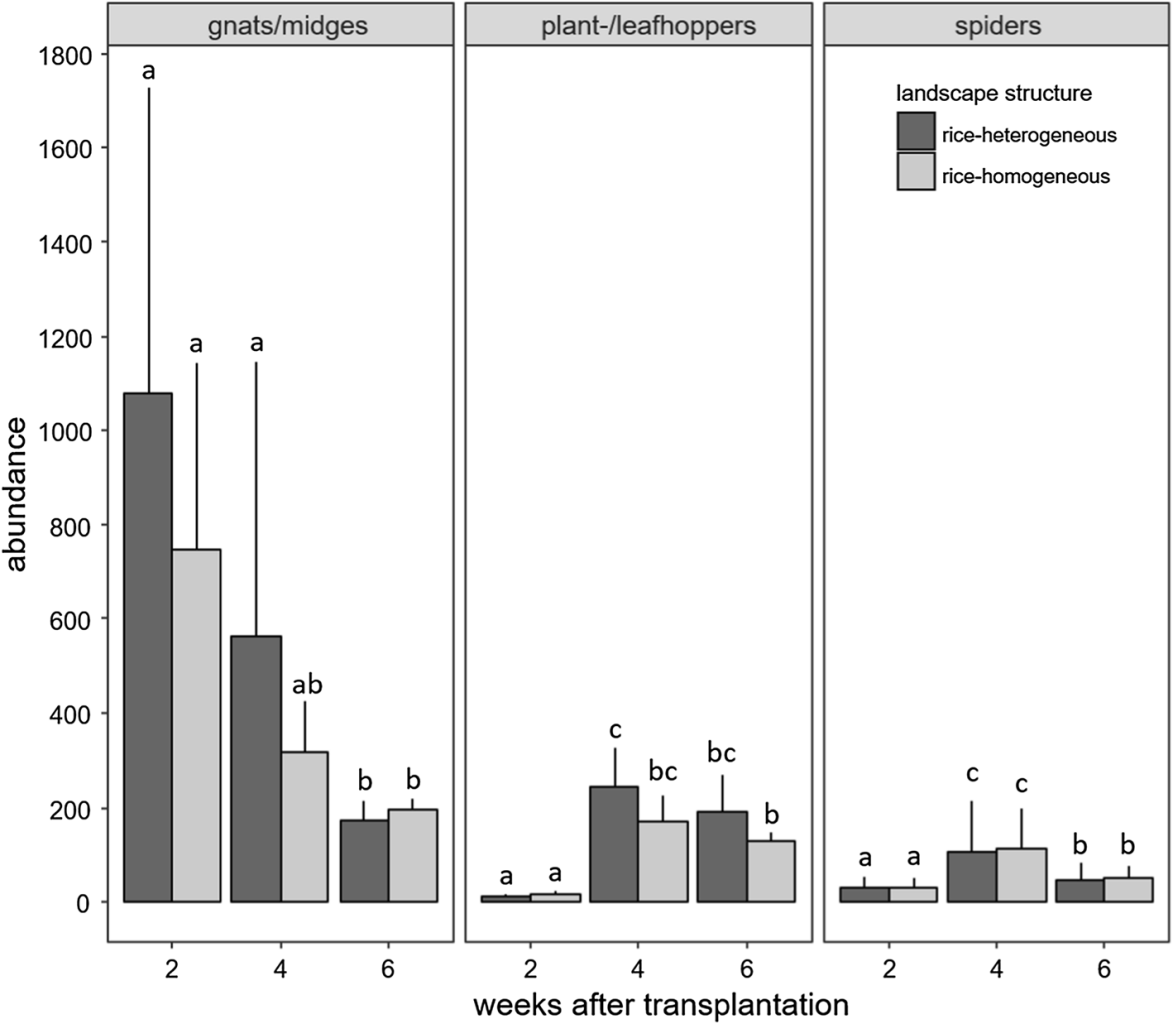
Abundance (mean ± SD) of adult gnats and midges, plant- and leafhoppers, and spiders 2, 4 and 6 weeks after transplantation of rice seedlings into the field in rice-heterogeneous and rice-homogeneous landscapes. Means not sharing the same letter differ significantly (Tukey’s HSD test, *p* < 0.05)

The abundance of adult gnats and midges differed significantly between sampling dates, but the effect also varied with landscape structure with densities on rice-heterogeneous fields exceeding those on rice-homogeneous fields by 30.6% on average (*F* = 43.1, *p* < 0.001 for date × structure; *F* = 29.5, *p* < 0.001 for date; Fig. 1, Online Resource 1). We found high abundances of gnats and midges 2 weeks after rice transplantation, averaging 1077 ± 653 (mean ± SD) and 745 ± 396 individuals per sample in rice-heterogeneous and rice-homogeneous fields, respectively. By 4 weeks, the abundance decreased to approximately half and by the final sampling date, it declined to 15% and 25% of week two abundance.

Further, plant- and leafhopper abundance also differed significantly between sampling dates and landscape structures (*F* = 4.4, *p* < 0.001 for date × structure; *F* = 100.8, *p* < 0.001 for date, Online Resource 1); on average it was 30.7% higher in rice-heterogeneous than in rice-homogeneous fields with the difference being more pronounced later in the season (Fig. 1). In contrast to gnats and midges, 2 weeks after rice transplantation, the abundance of plant- and leafhoppers was low averaging 12 ± 4 and 14 ± 9 individuals per sample in rice-heterogeneous and rice-homogeneous fields, respectively. By week 4, they had increased strongly to an average of 243 ± 81 and 169 ± 55 individuals per sample, but in week 6, they declined by 20–25% in both rice-heterogeneous and rice-homogeneous fields.

Spider abundance also differed significantly between sampling dates (*F* = 286.4, *p* = 0.001 for date; Fig. 1, Online Resource 1), with the changes being similar in rice-heterogeneous and rice-homogeneous fields. Similar to plant- and leafhoppers, 2 weeks after rice transplantation, spider abundance was low, averaging 30 ± 21 individuals per sample. Then, by week 4, the abundance increased more than threefold to an average of 109 ± 97 individuals per sample, but by week 6, dropped to about half.

### Variations in δ^15^N values

**Fig. 2:**
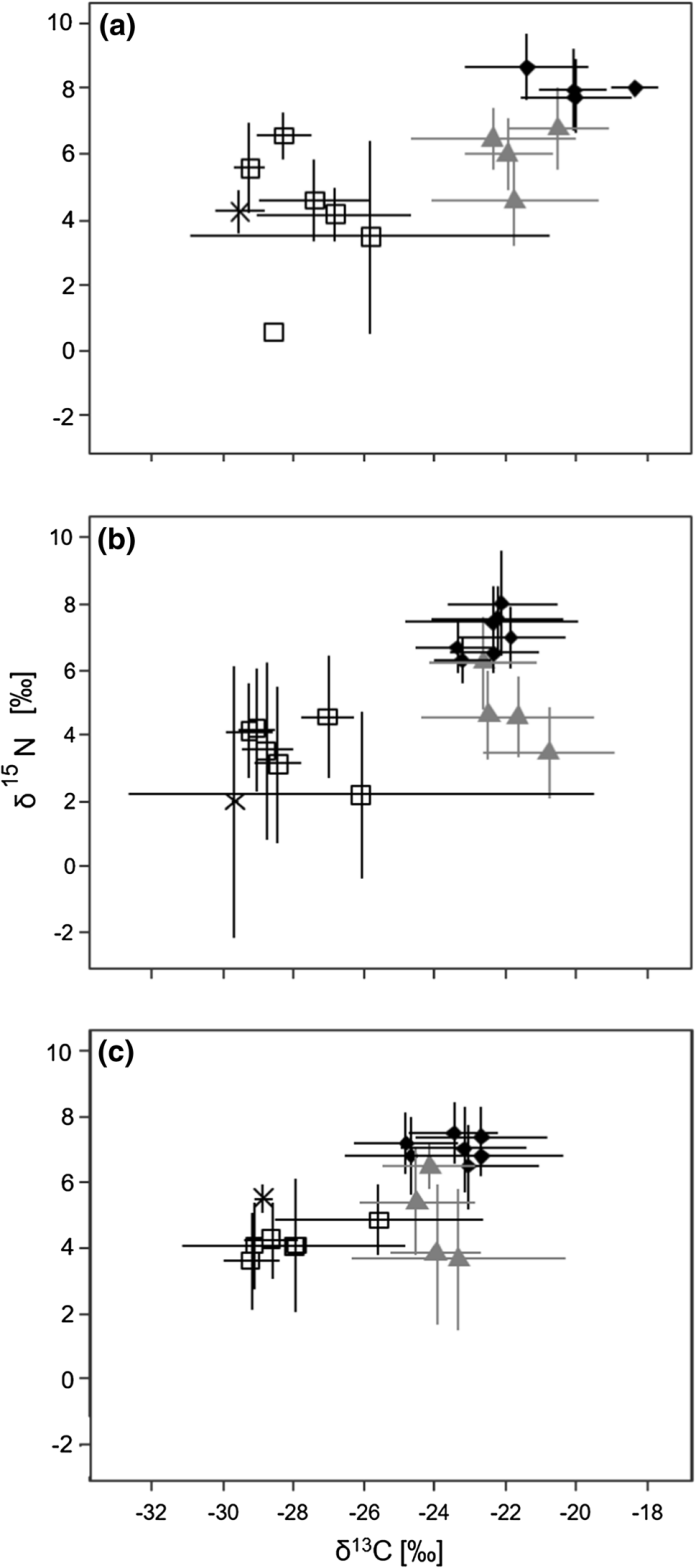
δ^13^C and δ^15^N values (mean ± SD) of rice (cross), spiders (filled circles), gnats and midges (triangles), plant- and leafhoppers (open squares) 2 (**a**), 4 (**b**) and 6 (**c**) weeks after transplantation of rice seedlings into the field

Two weeks after transplantation, rice had a δ^15^N value of 4.3 ± 0.7‰ (mean ± SD; Fig. 2, Online Resource 2). In larvae of gnats, δ^15^N values of 4.6 ± 1.4‰ were similar to those of rice. By contrast, δ^15^N values of midge larvae averaged at 6.5 ± 1.0‰. With an average of 6.0 ± 1.1‰, δ^15^N values of adult gnats exceeded those of the larvae; whereas, values of adult midges resembled those of the larvae and averaged 6.8 ± 1.3‰. δ^15^N values were low in plant- and leafhoppers with values ranging between 0.6‰ (*Recilia dorsalis*, Cicadellidae) and 6.6 ± 0.7‰ (planthopper nymphs). As predators, spiders were most enriched in ^15^N, with δ^15^N values ranging between 7.7 ± 0.6‰ in *Araneus inustus* (Araneidae) and 8.6 ± 1.0‰ in *Atypena adelinae* (Linyphiidae).

After 4 weeks, the δ^15^N value of rice decreased by 2.3‰ (Fig. 2). The range of δ^15^N values of plant- and leafhoppers narrowed and spanned from 2.2 ± 1.3‰ in leafhopper nymphs to 4.6 ± 1.9‰ in *Nephotettix nigropictus* (Cicadellidae). δ^15^N values of gnats varied more than in week 2, averaging 6.2 ± 1.4‰ and 3.5 ± 1.4‰ in adults and larvae, respectively. δ^15^N values of adult midges and larvae were again similar, averaging 4.6 ± 1.4‰ and 4.6 ± 1.2‰, respectively. In spiders, δ^15^N values remained high, but the range also narrowed to 6.3 ± 0.7‰ in *Dyschiriognatha hawigtenera* (Tetragnathidae) and 8.0 ± 1.6‰ in *Pardosa pseudoannulata* (Lycosidae).

Six weeks after transplantation, δ^15^N values of rice increased to 5.5 ± 0.4‰ (Fig. 2). In plant- and leafhoppers, δ^15^N values again varied little between 3.6 ± 1.5‰ in *R. dorsalis* and 4.9 ± 1.1‰ in *N. nigropictus*. δ^15^N values of adult gnats decreased to 3.8 ± 2.1‰, but gnat larvae stayed almost constant at 3.7 ± 2.1‰. By contrast, δ^15^N values of midge larvae and adults increased to an average of 5.4 ± 1.6‰ and 6.5 ± 0.7‰, respectively. The range of δ^15^N values in spiders changed little being lowest in *Tetragnatha virescens* (Tetragnathidae) with 6.5 ± 1.3‰ and highest in *Tetragnatha maxillosa* (Tetragnathidae) with 7.5 ± 0.9‰.

### Variations in δ^13^C values

**Fig. 3:**
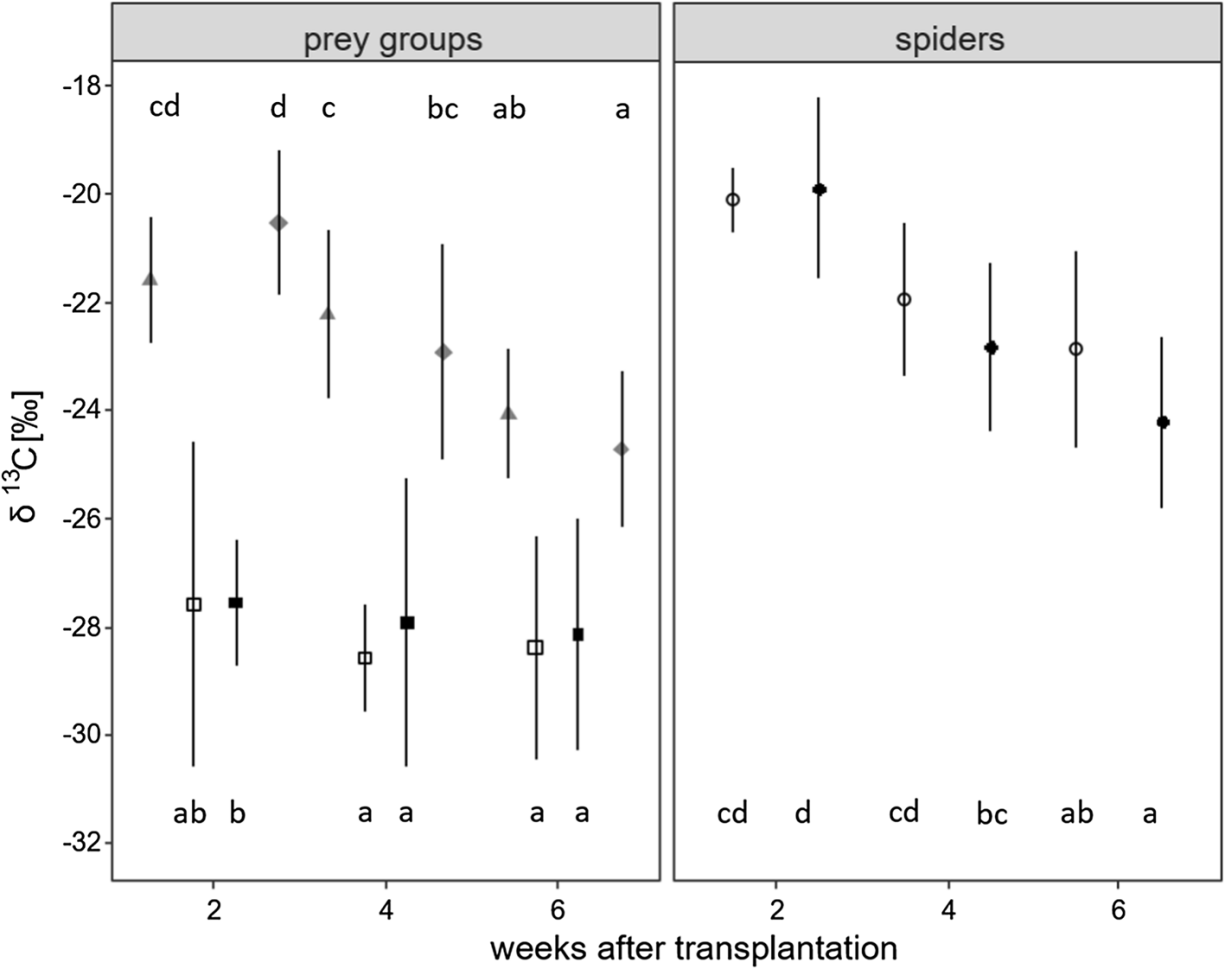
δ^13^C values of spiders (circles), gnats (triangles), midges (diamonds), plant- and leafhoppers (squares) in rice-heterogeneous (filled symbols) and rice-homogeneous (open symbols) fields 2, 4 and 6 weeks after transplantation of rice seedlings into the field pooled for species (mean ± SD). Grey symbols represent groups pooled for rice-heterogeneous and rice-homogeneous fields. Means not sharing the same letter differ significantly (Tukey’s HSD test, *p* < 0.05)

δ^13^C values of both gnats and midges declined from 2 to 4 to 6 weeks after rice transplantation, with the decline in midges being significant between each sampling date, whereas in gnats only weeks 2 and 6 differed significantly (*F*_2,55_ = 3.2, *p* = 0.0475 for date × species; Fig. 3, Online Resource 1).

**Fig. 4:**
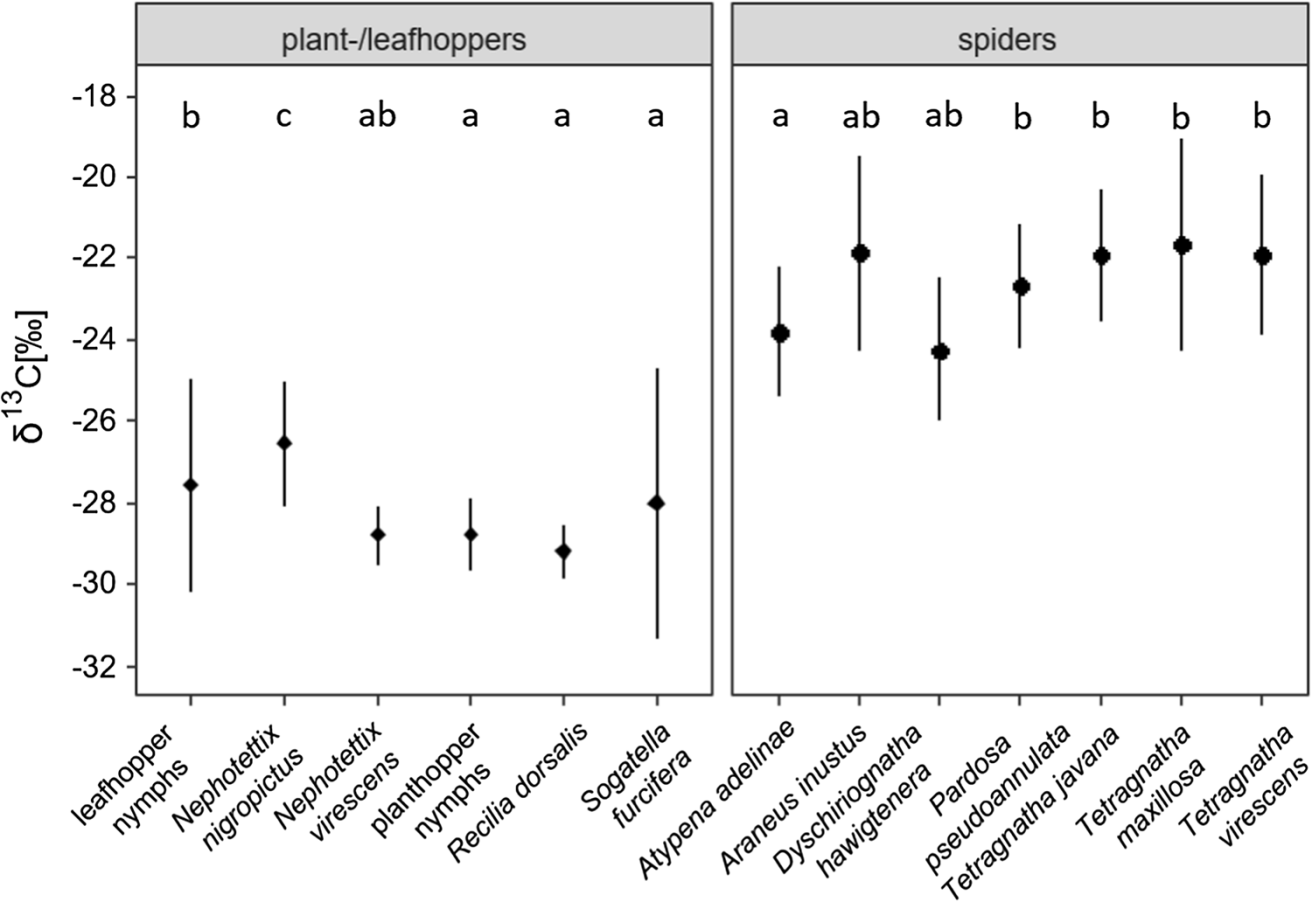
δ^13^C values of plant- and leafhopper species (Delphacidae: planthopper nymphs, *S. furcifera*; Cicadellidae: leafhopper nymphs, *N. nigropictus*, *N. virescens*, *R. dorsalis*) and nymphs and spider species (Linyphiidae: *A. adelinae*; Araneidae: *A. inustus*; Tetragnathidae: *D. hawigtenera*, *T. javana*, *T. maxillosa*, *T. virescens*; Lycosidae: *P. pseudoannulata*) pooled for sampling dates and rice-heterogeneous and rice-homogeneous fields (mean ± SD). Means not sharing the same letter differ significantly (Tukey’s HSD test, *p* < 0.05)

δ^13^C values of plant- and leafhoppers differed significantly between sampling dates, but the effect depended on landscape structure (*F*_2,122_ = 4.1, *p* = 0.0189 for date × structure, Online Resource 1). δ^13^C values generally declined later in the season, with the decline being more pronounced in rice-heterogeneous than rice-homogeneous fields (Fig. 3). Further, δ^13^C values of plant- and leafhoppers varied significantly between species with the differences being independent of date and structure (*F*_5,122_ = 24.0, *p* < 0.0001 for species; Fig. 4, Online Resource 1). However, differences were mainly due to the cicadellid *N. nigropictus*; δ^13^C values of this species significantly exceeded those of each of the other species.

As compared to plant- and leafhoppers, δ^13^C values of spiders decreased more strongly with sampling date and the decline was more pronounced in rice-heterogeneous than in rice-homogeneous fields (*F*_2,109_ = 5.0, *p* = 0.0086 for date × structure; Fig. 3, Online Resource 1). Independent of sampling date and landscape structure, δ^13^C values of spiders differed significantly between species (*F*_6,109_ = 5.4, *p* = 0.0001 for species; Fig. 4, Online Resource 1). They were low in the linyphiid *A. adelina* and the tetragnathid *D. hawigtenera*, and high in the araneid *A. inustus*, the lycosid *P. pseudoannulata* as well as the three tetragnathids *Tetragnatha javana*, *T. maxillosa* and *T. virescens*.

### Contributions of gnats and midges, and plant- and leafhoppers to the diet of spiders

The contribution of gnats and midges, and plant- and leafhoppers to the diet of spiders changed significantly with sampling date (*F*_2,42_ = 310.9, *p* < 0.0001 for date; Table 1, Online Resource 1). The percentage of gnats and midges decreased from an overall mean of 62.8 ± 6.8% to 61.7 ± 3.1% to 57.8 ± 5.5% at the first, second and third samplings, respectively. In parallel, the percentage of plant- and leafhoppers increased from 37.2 ± 6.8 to 38.3 ± 3.1 to 42.2 ± 5.5%. The contribution of the two different prey groups to the diet of spiders did not vary with landscape structure, but in trend it varied among spider species (*F*_6‚42_ = 1.9, *p* = 0.0968 for spider species; Table 1, Online Resource 1); resembling the pattern in δ^13^C values; the contribution of plant- and leafhoppers to the diet of *D. hawigtenera* and *A. adelinae* (overall mean, pooled for sampling date 41.8 ± 7.4%) were higher than that in the other five species (overall mean, pooled for sampling date 38.1 ± 1.1%).

**Table 1:** Contribution (% ± SD) of gnats/midges and plant-/leafhoppers comprising the diet of spider species pooled landscape structure 2, 4 and 6 weeks after transplantation (WAT) during the rainy season 2012 (for calculation see methods)

Spider species	2 WAT			4 WAT			6 WAT		
	Gnats/midges	Plant/leafhoppers	± SD	Gnats/midges	Plant/leafhoppers	± SD	Gnats/midges	Plant/leafhoppers	± SD
*Atypena adelinae*	65.4	34.6	1.1	60.9	39.1	23.4	51.1	48.9	26.9
*Araneus inustus*	64.0	36.0	25.6	61.0	39.0	24.9	60.4	39.6	14.9
*Dyschiriognatha hawigtenera*	–	–	–	56.5	43.5	22.9	50.0	50.0	26.6
*Pardosa pseudoannulata*	–	–	–	65.2	34.8	23.9	65.6	34.4	23.9
*Tetragnatha javana*	66.6	33.4	27.0	62.3	37.7	22.7	57.8	42.2	26.9
*Tetragnatha maxillosa*	50.9	49.1	37.4	60.1	39.9	26.6	58.8	41.2	26.8
*Tetragnatha virescens*	67.2	32.8	25.9	65.6	34.4	22.2	60.6	39.5	26.3
Mean	62.8	37.2		61.7	38.3		57.8	42.2	
SD	6.8	6.8		3.1	3.1		5.5	5.5	

## Discussion

The arthropod communities of rice paddies are linked in a food web comprising two main compartments: one aquatic, based in flooded rice fields, and the other terrestrial, established with the transplantation of rice plants. Each compartment is formed by distinctive feeding linkages, but the terrestrial system is inextricably bound to the aquatic compartment by subsidies of emerging adult aquatic insects (Settle et al. 1996; Wilby et al. 2006). Changing prey availability during the cropping season results in marked changes in the terrestrial food web with plant- and leafhoppers becoming prey to terrestrial predators, in particular spiders, later in the season (Heong et al. 1991, 1992; Schoenly et al. 1996). These characteristics of the rice-paddy ecosystem were confirmed in this study by both population dynamics and SIA of the three most abundant arthropod groups, gnats and midges, plant- and leafhoppers, and spiders. The δ^15^N values of these taxa spanned about 9 δ units, from 0.56 to 9.68‰. Assuming a trophic level fractionation of 2.5‰ between plants and herbivores, and 3.4‰ between predators and animal prey (Vanderklift and Ponsard 2003), the rice-paddy food web comprised three–four trophic levels, aquatic polyphages (gnat and midge larvae from the aquatic compartment), herbivores (leafhopper and planthopper nymphs and adults), and predators (spiders: tetragnathids, araneids, linyphiids and lycosids).

### Aquatic polyphages

The abundance of gnats and midges markedly exceeded that of plant- and leafhoppers as well as spiders early in the rice-growing season. The fields studied were flooded during ploughing, levelling and fertilisation prior to transplanting rice seedlings until about 1 week before harvest. Thus, midges and gnats could finish their larval phase and emerge as adults early during rice cropping, possibly even before rice transplantation (Clement et al. 1977). Higher abundance in fields from rice-heterogeneous habitats in weeks 2 and 4 could be ascribed to the proximity of non-rice habitats which function as refuge from which fields can be recolonized more quickly after flooding compared to rice-homogeneous fields. Larvae of both gnats and midges are aquatic and live as decomposers on detritus and rice litter by grazing and filter feeding (Oliver 1971). Adult gnats live as ectoparasites on vertebrates and invertebrates (Papp and Darvas 1997), which potentially contributes to their enriched δ^15^N values compared to larvae. Low variation in δ^13^C values indicates nearly constant use of the same resources throughout the early cropping season. Adult midges do not feed and thus retain the larval stable isotope composition; in fact, δ^15^N values of midge adults and larvae were similar throughout the study period, decreasing only slightly in week 4. High early season δ^15^N values of midges indicate pre-season consumption of decaying plant material colonised by microorganisms, i.e., they are secondary rather than primary decomposers (Scheu and Falca 2000; Oelbermann and Scheu 2010). The pronounced changes in δ^13^C values of midges also suggest a wide range of food resources, including detritus and algae ingested by grazing or filter feeding (Oliver 1971; Settle et al. 1996; Henriquez-Oliveira et al. 2003). Early in the season, rice plants are small and provide little shade to the field, promoting algal growth. δ^13^C values of planktonic algae are typically less depleted than those of C3 plants, but may vary considerably. In well-aerated water bodies such as marine systems, they are in the range of − 20‰ (France 1995; Hambäck et al. 2016); algae from rice fields have δ^13^C values of − 22.9 ± 0.03‰ (N. Radermacher, unpubl. data), which is consistent with gnats in our study and only slightly lower than midges. As rice plants increase in height, they increasingly shade the water body of the rice field and take up more nitrogen (Roger 1996; Fernández-Valiente and Quesada 2004), which presumably hampered algal growth at our study sites and may have induced a shift by gnats and midges from algae to detritus-based resources formed from rice residues of previous cropping cycles (Johnson 1987; Galizzi et al. 2012). The decrease in δ^13^C by approximately 2‰ in larvae and 4‰ in adults over the study period is in accordance with the findings of Park and Lee (2006), and points to a dietary shift from predominantly algae early in the season to more rice detritus-based resources later (detrital δ^13^C values − 28.2 ± 0.12  ‰; N. Radermacher, unpubl. data).

### Herbivores

In tropical regions with asynchronous planting, like the study area, plant- and leafhoppers immigrate into rice fields from the surrounding vegetation throughout the cropping season, enabling planthopper females to start oviposition immediately after rice transplantation (Cook and Perfect 1985; Mollah et al. 2011). With an average egg development time of 8–11 days, plant- and leafhoppers are able to build up large populations within few weeks (Dyck et al. 1979). All three measures used in this study support early-season immigration and colonisation of rice fields from neighbouring plants. Abundance of plant- and leafhoppers significantly increased between 2 and 4 weeks after transplantation as immigrant-laid offspring matured. Results of the present study indicate that rice-heterogeneous landscapes, where rice fields are surrounded by gardens, grassland and forests, significantly increase colonisation of rice fields by plant- and leafhoppers suggesting that these habitats function as refuges for rice insect pest species during fallow periods (Bambaradeniya and Edirisinghe 2008). While rice-heterogeneous landscapes presumably favour fast build-up of pest populations, plant- and leafhoppers also quickly colonised rice fields in rice-homogeneous landscapes, with their abundance 4 and 6 weeks after rice transplantation only 30.4% and 33.9% lower than in rice-heterogeneous landscapes, respectively. Overall, the results support earlier findings that the arthropod community on rice fields changes in abundance and diversity during the cropping season (Heong et al. 1991; Schoenly et al. 1996; Wilby et al. 2006), with the decline in abundance of plant- and leafhoppers later in the season being likely due to predators, in particular spiders.

Planthoppers feed on basal plant parts, while leafhoppers prefer aerial parts, such as leaves and leaf sheaths, but nymphs and adults of both taxa recovered in this study are known to preferentially suck phloem sap of rice (Dale 1994; Lu and Heong 2009). The considerable variation in week 2 δ^15^N values reflects immigration from a variety of other vegetation, while the narrow range of δ^15^N values later in the season indicates generations of plant- and leafhoppers that fed exclusively on rice. In contrast to herbivores feeding on bulk plant material, δ^15^N values of phloem-feeding insects typically match those of their host plants (McCutchan et al. 2003; Oelbermann and Scheu 2010), as phloem-feeding requires high nitrogen use efficiency resulting in low fractionation of ^15^N in the consumer (Pinnegar et al. 2001; Vanderklift and Ponsard 2003).

δ^13^C values presented the most nuanced picture, with significant variation over time, by distance between the rice field and other vegetation, and between species. Differences were greatest 2 weeks after rice transplantation, presumably due to colonisation of the rice fields from neighbouring habitats containing C4 grasses. C4 grasses are typically enriched in δ^13^C caused by a different photosynthetic pathway resulting in distinct δ^13^C values compared to C3 grasses (Fry 2006). While all of the plant- and leafhopper species found here feed preferentially but not exclusively on rice, the species effect was driven in large by *N. nigropictus*, which has the widest diet of any herbivore species collected. Its diet includes C4 plants such as *Echinochloa colona* and *Polytrias indica* (Dale 1994; Caton et al. 2010), found among the ruderal C4 grasses along field margins (Fried et al. 2018) and known to be used as additional food resources by *N. nigropictus* (Dale 1994; Schoenly et al. 2010).

### Predators

Spiders were present in rice fields at low densities 2 weeks after rice transplantation and reached maximum abundance 4 weeks after transplantation. Above-ground growth of rice plants provides structural habitat complexity, known to be the major limiting factor for web building spiders (Cherrett 1964; Turnbull 1973; Rypstra 1983). Together with high prey availability, this presumably fostered the increased abundance of web building spiders including tetragnathids, araneids and linyphiids through week 4. Six weeks after rice transplantation, the abundance of spiders significantly decreased. This may have been due to food shortage, suggested by decreased abundance of both prey types, combined with less effective prey capture by web-building spiders due to the denser rice canopy as well as predation and cannibalism (Olive 1982; Nentwig 1982; Wise 1993; Foelix 2011).

The δ^15^N and δ^13^C values of most spiders closely matched those of gnats and midges, and less those of plant- and leafhoppers, suggesting that they heavily relied on adult gnats and midges as food resources throughout the cropping season. This supports earlier findings that energy subsidies from aquatic systems may substantially contribute to the nutrition of terrestrial generalist predators (Sanzone et al. 2003; Gratton et al. 2008; Dreyer et al. 2012), and this also is confirmed by our mixing models. In particular, early in the rice cropping season, the contribution of gnats and midges to spider nutrition considerably exceeded that of plant- and leafhoppers, which is consistent with earlier suggestions (Settle et al. 1996; Park and Lee 2006). However, mixing models also confirmed that terrestrial prey, i.e. plant- and leafhoppers, substantially contributed to the diet of spiders. Overall, therefore, the nutrition of generalist predators in rice fields resembles that of typical agricultural systems such as wheat fields where soil detritivores such as Collembola contribute to the diet of generalist predators including spiders, thereby increasing biological control of herbivore pest species (Scheu 2001; Snyder and Wise 2001; von Berg et al. 2010). Notably, as indicated by lower δ^13^C values later in the season, the contribution of terrestrial prey to the diet of spiders in rice-heterogeneous fields exceeded that in rice-homogeneous fields, but this was not shown in the results of mixing models. This may reflect the generally higher density of plant- and leafhoppers in rice-heterogeneous compared to rice-homogeneous fields. The higher contribution of terrestrial prey in rice-heterogeneous compared to rice-homogeneous fields as indicated by δ^13^C values suggests that rice-heterogeneous systems aggravate apparent competition between terrestrial herbivores and aquatic polyphages, where gnats and midges represent a donor-controlled spatial subsidy to spiders contributing to strengthening biological pest control (Polis et al. 1997; Henschel et al. 2001).

Stable isotopes revealed different diets at the species level with δ^15^N values indicating two trophic levels of spiders, first- and second-order predators. Similar δ^13^C values in the free-hunting lycosid *P. pseudoannulata*, the three web-building tetragnathids and the web-building araneid *A. inustus* suggest that the larger spider species predominantly fed on emerging gnats and midges. However, lower δ^15^N and δ^13^C values in *P. pseudoannulata* at week 6 indicate that terrestrial herbivore prey became increasingly important later in the season, as previously suggested (Ishijima et al. 2006; see above). Low δ^13^C values in the linyphiid species *A. adelinae* and especially the tetragnathid species *D. hawigtenera* in weeks 4 and 6, indicate that these species relied most heavily on terrestrial prey and these figures generally were confirmed by mixing models. High δ^15^N values typical of second-order predators suggest that intraguild predation contributes significantly to spider nutrition (McNabb et al. 2001; Rickers et al. 2006), but this may be reduced by habitat complexity (Langellotto and Denno 2006; Sigsgaard 2007). In fact, maximum δ^15^N values in week 2 suggest that intraguild predation and cannibalism were more prevalent early in the cropping season; whereas, growth of rice plants forming more complex habitats resulted in a decrease in δ^15^N values in weeks 4 and 6. Notably, in week 2, δ^15^N values were highest in the linyphiid *A. adelina* suggesting that, despite its small body size, this species fed heavily on other predators including conspecifics, which is confirming earlier findings on linyphiid spiders (Vanacker et al. 2004; Park and Lee 2006). Although they do build webs, linyphiid spiders are also known to forage by hunting prey, thereby increasing their prey spectrum (Alderweireldt 1994; Uetz et al. 1999).

## Conclusion

Changing prey availability during the cropping season resulted in spiders initially consuming insects emerging out of the aquatic system, then shifting gradually to terrestrial plant- and leafhopper prey later in the season, particularly in rice-heterogeneous fields. δ^15^N and δ^13^C values of predators follow those of aquatic polyphages, while simultaneously converging toward herbivore values. This suggests that terrestrial food resources became increasingly important for both aquatic polyphages and predators. In the present study, nearby gardens, grassland and forests functioned as refuge from which plant- and leafhoppers colonised rice fields, thereby increasing the availability of terrestrial herbivore prey for spiders, as indicated by lower δ^13^C values of spiders in fields with more heterogeneous landscapes later in the cropping season. Prey preference, however, appears to depend on spider species of which larger web-builders and free hunters feed more on aquatic prey compared to smaller web-building/wandering species feeding more on terrestrial prey.

Overall, our results suggest that generalist predators of tropical rice-paddy fields are sustained by three different carbon sources. Early in the season, they predominantly rely on carbon fixed by algae of the water body of rice fields (incorporated via gnats and midges); whereas later in the season, they predominantly rely on legacy carbon from previous growing cycles (incorporated via gnats and midges) as well as rice carbon of the current season (incorporated via herbivore prey). Alternative prey out of the aquatic system appears to be of paramount importance for fostering biological control of rice insect pest species. Further research, including experimental approaches investigating crop residue management practices, are necessary to elucidate potentials of enhanced biological control. Management strategies leaving more crop residue in the fields are likely to increase the availability of gnats and midges from submersed rice fields, and therefore strengthen efficiency of biological control of rice insect pests. By contrast, the use of insecticides, particularly early in the season, reduces the availability of alternative prey and may critically compromise biological control of rice insect pest species by natural enemies in rice field ecosystems.

## Electronic supplementary material

Below is the link to the electronic supplementary material.

Supplementary material 1 (XLSX 15 kb)

Supplementary material 2 (XLSX 21 kb)
